# Functional imaging of the interaction between gut microbiota and the human host: A proof-of-concept clinical study evaluating novel use for 18F-FDG PET-CT

**DOI:** 10.1371/journal.pone.0192747

**Published:** 2018-02-15

**Authors:** Ben Boursi, Thomas J. Werner, Saeid Gholami, Sina Houshmand, Ronac Mamtani, James D. Lewis, Gary D. Wu, Abass Alavi, Yu-Xiao Yang

**Affiliations:** 1 Division of Gastroenterology, Department of Medicine, Perelman School of Medicine at the University of Pennsylvania, Philadelphia, Pennsylvania, United States of America; 2 Center for Clinical Epidemiology and Biostatistics, Perelman School of Medicine at the University of Pennsylvania, Philadelphia, Pennsylvania, United States of America; 3 Abramson Cancer Center, University of Pennsylvania, Philadelphia, Pennsylvania, United States of America; 4 Tel-Aviv University, Tel-Aviv, Israel; 5 Department of Oncology, Sheba Medical Center, Tel Hashomer, Ramat-Gan, Israel; 6 Department of Nuclear Medicine, Perelman School of Medicine at the University of Pennsylvania, Philadelphia, Pennsylvania, United States of America; National Institute for Agronomic Research, FRANCE

## Abstract

Recent data comparing germ-free to conventionally-raised mice demonstrated that energy homeostasis of colonocytes is dependent on gut microbiota through regulation of short chain fatty acids (SCFA) production and glucose utilization. We sought to evaluate 18F-FDG PET-CT as a novel technique for functional imaging of alterations in glucose metabolism as a result of the interaction between the gut microbiota and the human host. We conducted a prospective study in healthy humans that underwent 18F-FDG PET-CT and sampling of the gut microbiota before and after orally administered broad-spectrum antibiotics. The primary outcomes were total and regional physiologic colonic 18F-FDG uptake (measured as the mean and max standardized uptake values [SUVmean and SUVmax]). The study demonstrated significant increases in physiologic colonic 18F-FDG uptake in all study participants following antibiotic treatment and a 4-5log reduction of gut bacterial load. The mean increase in SUVmax was 0.63±0.37 SD (p = 0.004) and the median increase was 0.42 with an IQR of 0.40–0.81. The mean increase in SUVmean was 0.31±0.24 SD (p = 0.01) and the median increase was 0.41 with an IQR of 0.06–0.55. A likely explanation for this phenomenon is a shift in colonocyte metabolism to glycolysis due to a shortage of SCFA.

## Introduction

The gut microbiota is regarded by many as a distinct organ within the human body with a central role in metabolism, immunity, and inflammation [*1*]. Conditions such as obesity [*2–3*] and diabetes [*4–5*], auto-immune and allergic diseases [*6–9*], inflammatory bowel disease [*10*] and cancer [11*–15*] have been previously associated with alterations in the composition of the gut microbiota, referred to as dysbiosis. While recent advances in high throughput sequencing techniques together with the development of computational tools have enabled investigators to determine the composition of complex microbiota communities with unprecedented precision, to date there are no clinical tools to directly demonstrate the interaction between the gut microbiota as a whole and the human host. The gut microbiota is required for energy homeostasis in colonocytes, and short chain fatty acids (SCFA), produced by the gut microbiota mainly from the fermentation of undigestible carbohydrates, serve as the main nutrient for colonocytes [16–*18*]. Previous *in vitro* and *in vivo* studies [*18*] demonstrated a shift in colonic metabolism from SCFA to glycolysis in germ-free compared to conventionally-raised mice. We recently demonstrated, both in mice and humans, that the use of a combined protocol of two antibiotics (vancomycin and neomycin taken for 3 consecutive days) and Polyethylene glycol (PEG) on the second day reduces both culturable bacteria and 16S gene copy number by approximately 4–5 logs, creating a gut microbial environment that more closely mimics a germ-free mouse [*19*].

The uptake of 18F-FDG by tissues is a marker for the tissue uptake of glucose, which in turn is closely correlated with certain types of tissue metabolism. After 18F-FDG is injected into a patient, a PET scanner can form two-dimensional or three-dimensional images of the distribution of 18F-FDG within the body.

Positron emission tomography with 2-deoxy-2-[fluorine-18]fluoro- D-glucose integrated with computed tomography (18F-FDG PET-CT) uses a radioactive fluorodeoxyglucose (FDG) as a marker for the tissue uptake of glucose in order to trace glucose metabolism. The PET scanner form images of 18F-FDG distribution within the body. The biological principle behind 18F-FDG PET means that it can potentially be used as a novel method for functional imaging of the host-microbiota interaction by capturing the change in tissue metabolism following changes in gut microbiota load and composition.

The aim of the current study was to determine whether physiologic colonic 18F-FDG uptake can serve as a functional imaging tool capable of capturing the colonic metabolic shift occurring in the human host following reduction in gut bacterial load (required for the production of SCFA). This aim was investigated using a prospective clinical trial which compared the colonic 18F-FDG uptake in healthy humans before and after significantly reducing gut microbiota bacterial load via the administration of broad-spectrum antibiotics and a PEG purge. This clinical trial was embedded within a parent study (the FARMM study) which investigated the effect of diet and antibiotics on human gut microbiome and metabolomes [*19*].

## Materials and methods

### Study design

**Fig 1:**
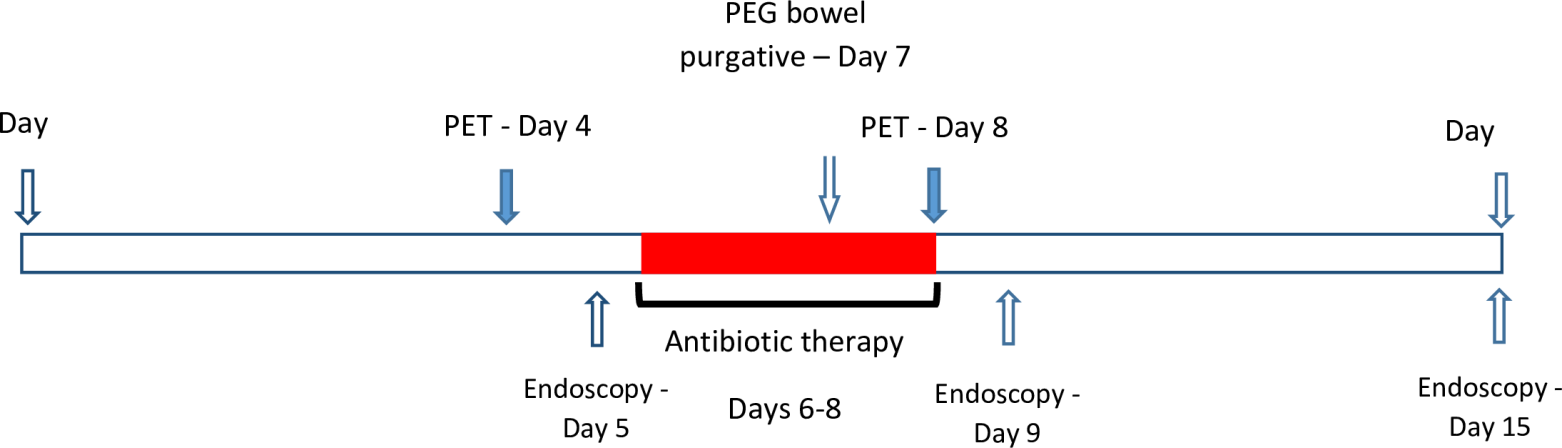
Timing of bacterial eradication, PET imaging and endoscopy.

We conducted a sub-study embedded within a parent study, the FARMM study [*19*]. FARMM is a randomized controlled feeding experiment examining the effect of diet and antibiotics on the composition of stool microbiome and fecal and plasma metabolome of 30 healthy volunteers age 18–60 years who were sequestered at the Clinical and Translational Research Center at the University of Pennsylvania for the duration of the study. Individuals with inflammatory bowel disease (IBD), celiac disease, irritable bowel syndrome (IBS) and those who took antibiotics in the 6 months prior to study initiation were excluded. As part of the FARMM protocol subjects provided fecal samples and underwent sigmoidoscopy with rectal mucosal biopsy prior to initiating a controlled diet. All participants were followed for 15 days. All participants were treated with broad-spectrum antibiotics daily for 3 days (days 6–8) (i.e., vancomycin 500mg orally every 6 hours and neomycin 1000mg orally every 6 hours). Of note, these antibiotics are largely non absorbable from the gastrointestinal tract and have no known effect on the colonic epithelium [*20,21*]. On day 7, the participants also consumed 4L of polyethylene glycol (PEG) based bowel purgative (GoLytely®). Stool samples were collected daily. Rectal biopsies were obtained at days 5, 9, and 15 **(Fig 1)**. The current study included 7 of the 30 participants in the FARMM study who consented to undergoing PET-CT before and after the antibiotic treatment. In a previous publication from the FARMM study we demonstrated a 4-5-log reduction in bacterial load following antibiotic administration using 16S rRNA gene copy PCR [*19*]. The study was approved by the Institutional Review Board at the University of Pennsylvania.

### 18F-FDG PET-CT imaging

**Fig 2:**
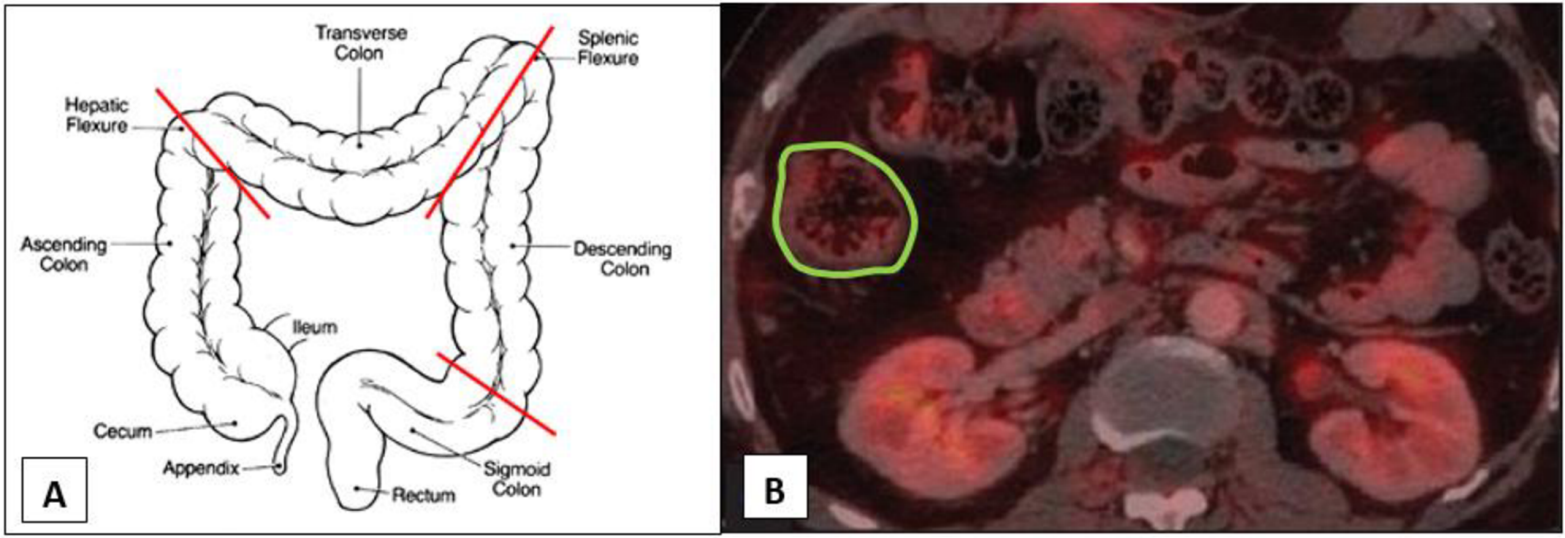
Definition of colonic regions and example of a typical region of interest (ROI). (A) Proximal colon defined from the cecum to the hepatic flexure. Transverse colon defined from the hepatic to the splenic flexure. Descending colon defined from the splenic flexure to the left iliac crest. Recto-sigma defined from the left iliac crest to the anus. (B) A typical region of interest (ROI) definition in the proximal colon.

PET scans were performed on days 4 and 8 of follow-up and at the same time of the day. Subjects were fasting for 4 hours prior to FDG injection. Blood glucose levels were measured before the administration of FDG and did not exceed 200 mg/dL at the time of injection. PET imaging scans of the abdomen and pelvis were performed 60 minutes after injection of 5 mCi 18F-FDG. CT images were acquired using a low-dose technique (120kV peak and 30–50 mAs) for attenuation correction of PET images. All images were performed using the same scanner (Philips, Ingenuity TF PET-CT). **18F-FDG PET-CT image analysis:** Image data was analyzed using a dedicated image visualization and analysis software (OsiriX MD 64bit version). In each person, Regions of interest (ROIs) were drawn manually on each transaxial slice around the outer boundaries of the four colonic regions (cecum and ascending, transverse, descending, and rectosigmoid) (**Fig 2**). In addition, we defined ROI for abdominal subcutaneous tissue in three transaxial slides below the lower pole of the right kidney for adjustment purposes as a reference tissue. The drawings were done according to anatomic definitions using CT images. In case of overlap between areas of colon and small bowel, the areas were excluded from the ROIs. Uptake was measured using standardized uptake value (SUV, the concentration of tracer measured by PET in the target tissue divided by the activity injected divided by body weight) in order to remove variability caused by the amount of 18F-FDG injected and differences in patient size. A single investigator (BB) read the scans and was blinded to both subject and timing of the image (before or after antibiotics).

### Primary outcome

The primary outcomes were average and maximum colonic 18F-FDG uptake (SUVmean and SUVmax) measured within all ROIs of the relevant colonic region of each individual, both before and after antibiotic treatment.

### Statistics

The primary analysis compared SUVmean and SUVmax, both in the entire colon and in specific colonic regions, before and after antibiotic treatment using paired t-test. The results were adjusted for abdominal subcutaneous FDG uptake using linear regression. All statistical analyses were performed using STATA 13 (STATA Corp. College Station, TX, USA).

## Results

Among the 30 participants of the FARMM study 7 individuals took part in the prospective PET sub-study.

**Fig 3:**
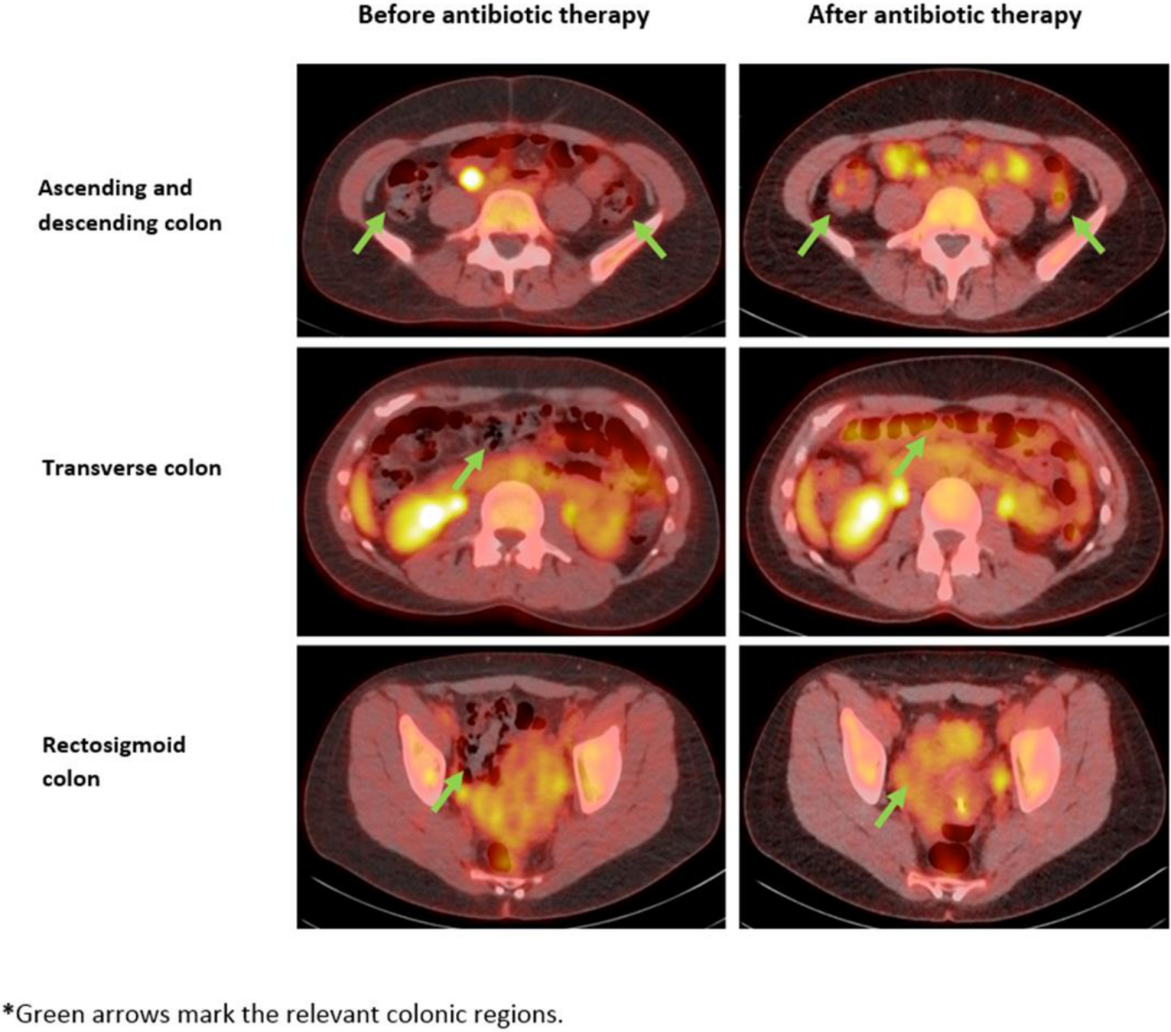
18F-FDG PET uptake in different colonic regions before and after antibiotic therapy in the first individual enrolled to the study.

**Table 1** presents colonic 18F-FDG uptake among individuals in the prospective study before and after antibiotic therapy, both as total colonic SUVmax and total colonic SUVmean. Both values increased in all patients after antibiotic treatment. The change in uptake was the highest in the cecum and ascending colon (**Table 1**). The mean increase in SUVmax was 0.63±0.37 SD (p = 0.004) and the median increase was 0.42 with an IQR of 0.40–0.81. The mean increase in SUVmean was 0.31±0.24 SD (p = 0.01) and the median increase was 0.41 with an IQR of 0.06–0.55. The absolute change in SUVmax was the largest in individuals with the lowest SUVmax before antibiotic administration. **Table 2** demonstrated the increase in total colonic and regional SUVmax and SUVmean following antibiotic therapy. **Fig 3** presents PET images of 18F-FDG uptake in different colonic regions before and after antibiotic therapy in the first individual that was enrolled.

**Table 1:** Physiologic colonic 18F-FDG uptake among individuals before and after antibiotic therapy.

	Before Abx.			After Abx.			Delta SUVmax (SUVmax before Abx. –SUVmax after Abx.)
	Cecum and ascending colon SUVmean	Total SUVmax	Total SUVmean	Cecum and ascending colon SUVmean	Total SUVmax	Total SUVmean	
Subject 1	0.518	0.976	0.591	1.036	1.779	1.140	0.803
Subject 2	0.656	1.529	0.679	1.120	2.845	1.088	1.316
Subject 3	0.958	1.834	0.922	1.457	2.250	0.956	0.416
Subject 4	1.929	1.809	1.204	1.794	2.220	1.278	0.411
Subject 5	1.130	1.735	1.046	1.616	2.009	1.105	0.274
Subject 6	0.996	1.466	0.832	1.990	2.271	1.387	0.805
Subject 7	0.992	2.084	1.063	2.351	2.486	1.551	0.402

**Table 2:** Change in total colonic and regional SUVmean and SUVmax among individuals in the FARMM study before and after antibiotic therapy.

	Before Abx.	After Abx.	P-value
**Entire colon**			
**SUVmean**	0.99 (0.73–1.24)	1.22 (1.06–1.38)	0.03
**SUVmax**	1.75 (1.36–2.13)	2.25 (1.99–2.52)	0.01
**Cecum and ascending colon**			
**SUVmean**	1.13 (0.70–1.56)	1.57 (1.19–1.96)	0.04
**SUVmax**	1.94 (1.21–2.67)	3.03 (2.37–3.69)	0.01
**Transverse colon**			
**SUVmean**	0.86 (0.66–1.06)	0.99 (0.84–1.14)	0.14
**SUVmax**	1.57 (1.20–1.95)	1.78 (1.61–1.95)	0.14
**Descending colon**			
**SUVmean**	1.03 (0.78–1.27)	1.14 (1.01–1.27)	0.19
**SUVmax**	1.75 (1.36–2.14)	1.98 (1.68–2.28)	0.16
**Rectosigmoid**			
**SUVmean**	1.05 (0.79–1.31)	1.28 (1.03–1.52)	0.05
**SUVmax**	1.84 (1.37–2.31)	2.40 (1.76–3.05)	0.03

## Discussion

We demonstrated an increase in physiologic colonic 18F-FDG uptake in all study participants following antibiotic treatment that results in a 4-5log reduction in gut bacteria load. Using 18F-FDG PET scans, we visually demonstrated for the first time in healthy human subjects a change in colonic glucose metabolism resulting from a decrease in bacterial load following treatment with broad spectrum antibiotics. These results are supported by both *in vitro* and *in vivo* data showing a shift in colonic metabolism in germ-free mice from SCFA (mainly butyrate), normally produced by the gut microbiota, to glycolysis.

Of note, the degree of change in physiologic colonic 18F-FDG uptake was inversely correlated to the initial uptake before antibiotic treatment (**Fig 2**). Thus, individuals with the lowest initial uptake, reflecting normal diverse microbiota that allows metabolism based on SCFA, had the highest increase in SUV following antibiotic treatment and resulting decrease in bacterial load and transition to glycolysis-based metabolism.

The current results also suggest a possible explanation for a clinical observation, previously described in the literature, showing diffuse colonic 18F-FDG PET uptake in diabetic patients that are treated with metformin [*22,23*]. This uptake can be reduced if the medication is stopped 48 hours prior to PET imaging [*24–26*]. Metformin, an oral hypoglycemic medication, is known to activate the AMP-activated protein kinase (AMPK) pathway, a key regulator of cell metabolism, promoting glycolysis and generation of ATP while inhibiting anabolic pathways, thus increasing 18F-FDG avidity [*27–29*]. This change is prominent in the colon since it is a metabolically active tissue that under normal conditions utilizes SCFA as an energetic source. This alteration observed among metformin users is similar to the metabolic shift in gut metabolism from SCFA to glycolysis following reduction in colonic bacterial load. Of note, some of the therapeutic effects of metformin were recently shown to be mediated through change in the gut microbiota [*23*].

The study had the advantage of evaluating healthy subjects without recent antibiotic exposure that received a fixed diet under medical supervision for the study duration. All PET scans were performed during the same time of day and in the same scanner. All images were read by the same physician that was blinded to both patient and timing of the test in relation to antibiotic treatment. Since analysis was performed within individuals as a paired t-test, comparing uptake before and after antibiotics, there was minimal risk for additional confounding as an explanation for study results. Furthermore, in contrast to previous studies [*30*], we used antibiotics with intraluminal activity and no known anti-inflammatory activity reducing nonspecific uptake.

The current study had several limitations. Our study had a small sample size. However we were still able to detect a consistent increase in 18F-FDG uptake in all colonic regions (although not significant for transverse and descending colon) and correlate this increase with previously described biological pathways [*18*]. The normal gastrointestinal tract exhibits low physiologic 18F-FDG uptake [*31*]. Suggested sources for the uptake include mucosal metabolic activity, smooth muscle contractions, and glandular secretions [*30*]. Additional studies suggested that this uptake may also occur due to intra-luminal activity. Since gut microbes are one of the main luminal components, it was proposed that the gut microbiota may play an additional role in concentrating luminal 18F-FDG [*32*]. A single study demonstrated suppression of mean background 18F-FDG uptake in individuals without known colonic disease following pretreatment with the antibiotic rifaximin for 2 days [*30*]. However since rifaximin has an additional anti-inflammatory activity, it is possible that the decrease in uptake was secondary to decrease in inflammation. So far, no study has been able to measure intra-luminal or fecal 18F-FDG or suggest a mechanism for possible 18F-FDG transition from the intra-vascular compartment to the gut lumen. In the current study we demonstrated not only an increase in total colonic 18F-FDG uptake but also an increase in each of the different colonic regions, further supporting a diffuse metabolic effect rather than a local effect (such as muscle contraction or inflammation) as an explanation for the results. Finally, we cannot rule out possible direct effects of the antibiotic therapy or the PEG on the colonic mucosa as a possible explanation for the increased uptake, however since the PET imaging was done after this treatment it is a less likely explanation.

Although the current study measured both SUVmean and SUVmax and found similar statistically significant results for both measures, we recommend using SUVmax for future measurements of physiologic colonic uptake. While SUVmean includes the entire luminal uptake within the ROI which is mainly composed of air that has no 18F-FDG uptake, SUVmax consists largely of true mucosal uptake, increasing our ability to detect even minor changes.

In summary, we observed that reduction of colonic bacterial load by broad-spectrum antibiotics can be detected in humans by observing an increase in physiological colonic 18F-FDG uptake possibly due to a shift in colonocyte metabolism from lipolysis of SCFA to glycolysis. Therefore, colonic 18F-FDG uptake may represent a novel imaging approach to capturing the functional consequence of the microbiota-host interaction. This method may provide in the future a simple tool for early detection of a large number of diseases that are associated with change in the microbiota. Future studies are required in order to validate our results, evaluate the effect of specific diets on the microbiota and 18F-FDG uptake, and assess options of using different tracers, such as SCFA, as a way to image additional metabolic pathways. The described method views the microbiota as a distinct organ that communicates with its environment and affects multiple biological pathways. Using PET imaging allows us to study the functional aspects of microbiota-host interaction compared to genetic methods that focus on identification of specific bacterial phyla. Together these two methods are complementary and of utmost clinical significance.
